# Oral symptoms in dying nursing home patients. Results from the prospective REDIC study

**DOI:** 10.1186/s12903-024-03901-x

**Published:** 2024-01-25

**Authors:** Reidun K. N.M. Sandvik, Bettina S. Husebo, Geir Selbaek, Gunhild Strand, Monica Patrascu, Manal Mustafa, Sverre Bergh

**Affiliations:** 1 Department of Health and Caring sciences, Faculty of Health and Social Sciences, Western University of Applied Sciences, Haukelandsbakken 15, Bergen, N-5009 Norway; 2 Department of Global Public Health and Primary Care, Centre for Elderly and Nursing Home Medicine, University of Bergen, Bergen, Norway; 3 The Research Centre for Age Related Functional Decline and Diseases, Innlandet Hospital Trust, P.O. box 68, Ottestad, 2313 Norway; 4 The Norwegian National Centre for Ageing and Health (Ageing and Health), P.O. box 2136, Tønsberg, 3103 Norway; 5 Faculty of medicine, University of Oslo, Oslo, Norway; 6 Department of Geriatric Medicine, Oslo University Hospital, Oslo, Norway; 7 Department of Global Public Health and Primary Care, Neuro-SysMed Center, University of Bergen, Bergen, Norway; 8 Department of Clinical Dentistry, Faculty of Medicine, University of Bergen, Bergen, Norway; 9 Department of Automatic Control and System Engineering, Complex Systems Laboratory, University Politehnica of Bucharest, Bucharest, Romania; 10 Oral Health Centre of Expertise in Western Norway, Bergen, Norway

**Keywords:** Oral health problems, Palliative Care, Dementia, Nursing homes, Observational study

## Abstract

**Background:**

The mouth is a central organ for communication and fluid intake, also for dying nursing home patients. This study describes the prevalence and severity of oral symptoms from nursing home admission until the day of perceived dying and the day of death.

**Methods:**

A prospective, longitudinal cohort study including 696 patients who were admitted to 47 Norwegian nursing homes in 35 municipalities. During the first year of their stay, 189 died (27%), of whom 82 participants were assessed on the day they were perceived as dying and 134 on the day of death. Mouth care, nutrition, and bedsores were assessed with the Residents’ Assessment Instrument for nursing homes (RAI-NH) and palliative care (RAI-PC). Pain intensity was assessed with the Mobilization-Observation-Behaviour-Intensity-Dementia-2 Pain Scale (MOBID-2).

**Results:**

The proportion of patients with ≥ 6 oral symptoms increased from 16% when perceived as dying to 20% on the day of death (*P* = 0.001). On the day of death, xerostomia (66%), dysphagia (59%), and mastication problems (50%) were the most frequently observed oral symptoms. Only 16% received mouth care every hour and 12% were in pain during this procedure. Compared to people without dementia, those with a diagnosis of dementia at admission (*N* = 112, 86%) had xerostomia and mastication problems more frequently (50% vs. 73%; 32% vs. 56% (*P* = 0.038), respectively) on the day of death.

**Conclusions:**

The high extent of oral symptoms such as xerostomia, dysphagia, and mastication problems underline the need for systematic assessment and improved oral palliative care for dying nursing home patients with dementia.

**Trial registration:**

Clinicaltrials.gov NCT01920100 08/08/2013. First submission to BMC oral 15/03/2023.

## Introduction

Dementia has become increasingly prevalent with the rapid aging of the populations in both low- and high-income countries. Dementia affects 50 million people today, expected to triple to 152 million by 2050 [1] and is now the fifth leading cause of death worldwide [2]. Advanced age and progression of dementia are strongly associated with nursing home admission, which is also be the place where most of the affected persons die [3]. Dementia is a progressive and incurable condition that demands person-centred care and treatment [4]. Nursing home staff need to rely on proxy rating to obtain information on symptoms and needs when caring for persons with moderate to severe dementia. Adequate assessment is vital for the identification and treatment of symptoms. Unfortunately, people with dementia and dying patients may be unable to provide a self-report. This inability creates a substantial barrier for assessment and can place vulnerable patients at risk for under-recognition and under-treatment of symptoms when death is imminent [5]. To improve the quality of life and the quality of death and dying, palliative care has become a central goal at the end of life [6, 7]. The World Health Organization advocates for primary care services to incorporate palliative care that ensures adequate and timely care beneficial for all people with serious illnesses such as dementia [8]. This underlines the need for evidence-based, beneficial and equitable amenities to provide proper assessment and treatment of pain and burdensome symptoms, including mouth care, behavioural care resistance, and oral health problems as core elements [9, 10].

Oral health problems such as xerostomia (dry mouth), halitosis (bad smell), dysphagia (swallowing problems), gingival bleeding, plaque, caries, decayed teeth, and mucosal lesions are frequently described in nursing home patients [11]. Lack of specialized oral care training and skills in nursing home staff are two of the reasons for insufficient oral health [12, 13]. Our research group completed a questionnaire-based interview among health care staff about their oral care provision. Deputy nurses from 76 institutions (19 hospitals and 57 nursing homes), representing all Norwegians counties, answered. The study discovered a lack of oral care procedures in 25% of the Norwegian health care institutions, and that oral health problems were not recognized as important by 50% of the institutions [14]. A previous study revealed that among 325 nursing home patients, 61% of patients with their own teeth experienced at least one health problem originating in the mouth [15]. Patients with and without their own teeth suffered from xerostomia (41%) and dry, sore or cracked lips (34%). The high prevalence of xerostomia in the general nursing home population was also seen in a cross-sectional study including four long-term geriatric wards in France. In all, 37.5% of the 769 nursing home patients reported xerostomia, mainly caused by advanced age and anticholinergic medication use [16].

At the end of life, xerostomia may increase, caused by medication with anticholinergic side-effects, polypharmacy, dehydration, and a shift in breathing through the nose to breathing with an open mouth. Consequently, the dryness of the oral cavity may lead to symptoms such as bleeding spots, tongue inflammation, candida infection, and pain [17]. It is vital to note that the number of symptoms (severity) and the grade (intensity) of the symptoms seen in patients depend on the comprehensiveness of the assessment system used [18]. The severity of the oral symptoms, especially for xerostomia, thirst, bad smell and inability to speak is substantial at end of life [19]. Adding to this are the reports that oral status can be associated to survival length [20].

Oral health problems are assessed during the last week of life in 77% of patients with dementia, and while these assessments are associated with younger ages and women, very few studies exist on oral health problems in dying persons with dementia, leaving an information gap on symptom burden and management [21].

The aim of this study is to explore the oral symptom burden in dying nursing home patients, with or without dementia at admission. A secondary objective is to describe the symptom severity and treatment intensity on the day when the patient was perceived as dying by their closest caregiver and on the day of death.

## Method

### Design

The Recourse Use and Disease Course in dementia - Nursing Home (REDIC-NH) is a Norwegian longitudinal observational study including 696 patients admitted to any of the 47 nursing homes included in the study, representing 37 urban and rural municipalities [22]. Included patients were followed over time with biannual assessments until death. Caregivers working in the nursing homes did the assessments under the supervision of research nurses. The caregivers were trained as data collectors through a two-day training programme. The research nurses received a more comprehensive training programme and structured supervision by the study managers. Demographic data were retrieved from the patients’ medical records, whereas other variables were collected through structured interviews with the patients and their formal caregivers. Assessments were performed by the patients’ primary caregiver within four weeks after nursing home admission.

### Procedure

The REDIC-NH-End of life is a sub-study of the REDIC-NH study and comprises 189 patients included during baseline data collection between January 2012 and November 2014, who died between January 2012 and January 2015.

**Fig. 1:**
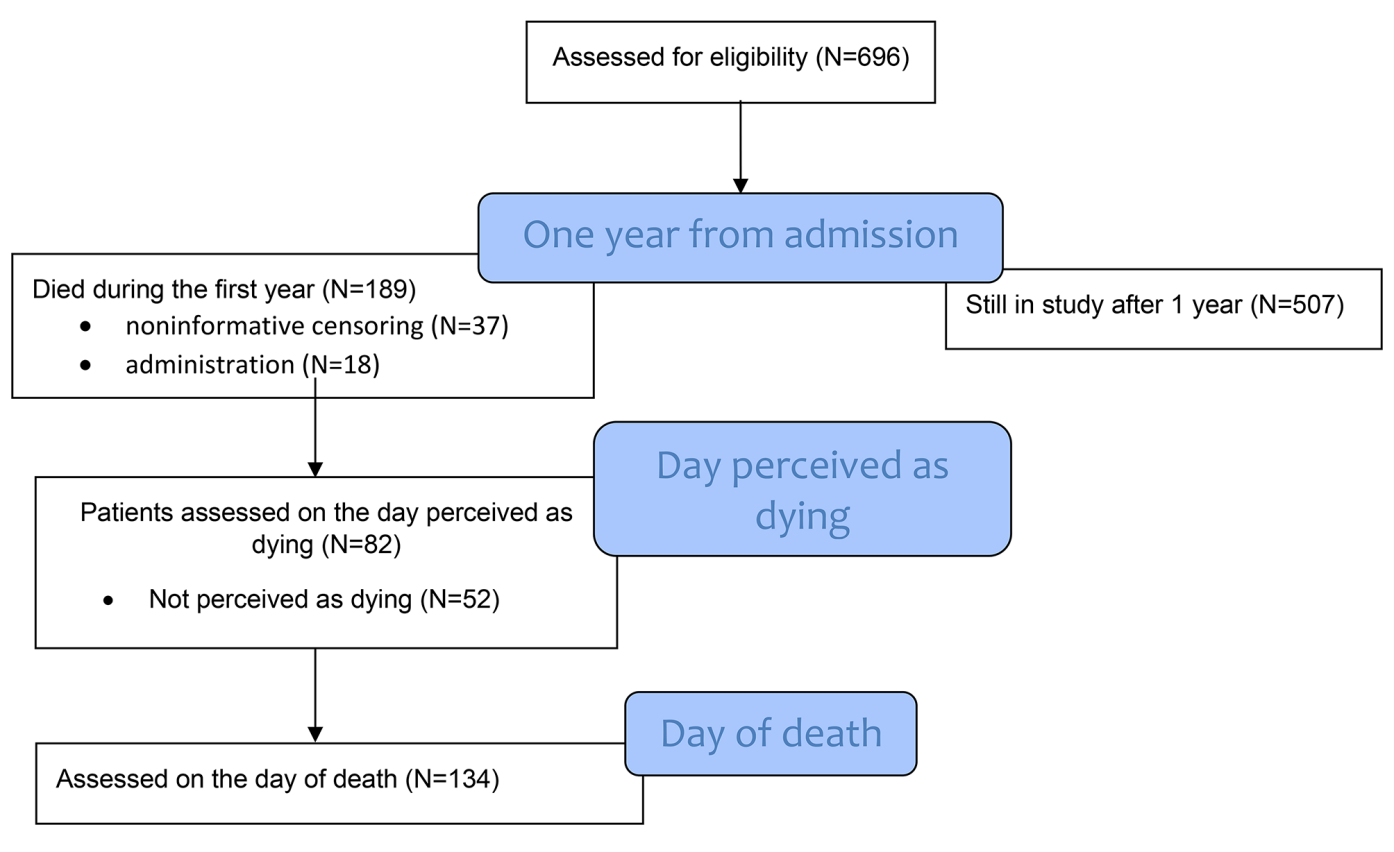
Overview of the study timeline

The REDIC-NH-End of life sub-sample was investigated for pain, symptoms, and end-of-life care treatment at baseline, on the day they were perceived as dying and on the day of death (Fig. 1). On the day when the patient’s primary caregiver, together with the responsible physician, perceived the patient as dying and on the day of death, the caregiver called the research nurse, and they performed an interview over the phone to ensure that this was a prospective study. The patient’s primary caregiver (registered nurses or licensed practical nurses), who provided daily care and had close contact with the patient, performed the assessments in collaboration with a research nurse.

### Participants

Patients were eligible when aged 65 years and older, or below 65 years and diagnosed with young onset dementia, admitted to long-term care in a nursing home, and expected to survive six weeks or longer. Participants (*N* = 696) were included at admission to a nursing home, 189 of which died within one year after admission (and no later than January 2015) and were included in this study. To obtain homogeneity, we excluded 37 patients who died shortly after admission. More details are given in our previous paper and Fig. 1 [4].

### Ethics

The Regional Committee for Medical and Health Research Ethics in Norway approved this study (2011/1738). The study was also registered at clinicaltrials.gov NCT01920100.

### Assessment tools

#### Baseline data collection

The degree of dementia was assessed with the Clinical Dementia Rating scale (CDR) [23]. The CDR contains six domains of cognitive and physical function (memory, orientation, judgment and problem solving, community affairs, home and hobbies, personal care). Each domain is scored as 0, 0.5, 1, 2, or 3. Based on an algorithm considering memory as the primary domain, a total score is derived with the same five values (0, 0.5, 1, 2, and 3), where 0 is no dementia, 0.5 is questionable dementia, and 1, 2 and 3 are mild, moderate, and severe dementia, respectively. Two of the authors (Geir Selbaek and Sverre Bergh), both psychiatrists with clinical and research expertise within geriatric psychiatry, diagnosed patients independently into: no cognitive impairment, mild cognitive impairment according to the Winblad criteria, and dementia according to the ICD-10 criteria [22, 24]. A third expert was called in to resolve cases of discrepancy.

We used the Residents’ Assessment Instrument for Nursing Homes (RAI-NH), which is a structured assessment tool for care status consisting of 18 sections such as cognition and physical function. We included the sections for oral health problems, nutrition, and bedsores [25].

Pain was assessed using the Mobilisation-Observation-Behaviour-Intensity-Dementia-2 Pain Scale (MOBID-2) [26]. The MOBID-2 scale is a behaviour-based observational pain tool containing ten items to assess the prevalence of pain that might be related to the musculo-skeletal system or internal organs, head, and skin. Pain intensity is provided as a total score on a numeric rating scale from 0 to 10 (0 is no pain; 10 is the worst possible pain). In the analyses, we included the total score and the item for oral pain. Appetite was assessed with the Edmonton Symptom Assessment System (ESAS), a tool including nine symptoms (fatigue, drowsiness, nausea, pain, dyspnoea, depression, anxiety, poor appetite, and well-being) [27]. ESAS is a continuous numeric rating scale ranging from 0 to 10. Previous studies have confirmed the symptom cut off scores at 0–2 (mild), 3–6 (moderate) and 7–10 (severe) [28].

#### Imminent dying

Identification of the last days and hours of life is vital for optimized management in this period. We therefore assessed the day perceived as dying, which is the day a team of nurses and a physician with close knowledge of the patient declare that death is imminent.

On the day the patient was perceived as dying and on the day of death, patients were assessed using the Residents’ Assessment Instrument for Palliative Care (RAI-PC). This tool includes eight domains, from which we selected oral care, nutrition and nutritional status (including cachexia), and bedsores [29]. Fluid intake was measured in cups (approximately 250 ml per cup). To further register the most important oral symptoms in dying people with dementia, the dentist (Gunhild Strand) developed a dichotomous scale containing the following indicators: xerostomia, mucus, crusts, halitosis, bleeding, sores, candidiasis, other infections, pain at rest, pain during oral cleansing, masticatory problems, swallowing difficulties, and reduced ability to speak. Each item was scored as present or not present (1 or 0). Oral hygiene was assessed according to oral care frequency, possession of own teeth, and the use of a prosthesis.

### Statistical analyses

Continuous variables were described by their means and standard deviations (SD), and categorical variables by proportions. Logistic regression models were built for binary outcomes and linear regression models for continuous outcomes to investigate the possible associations between variables at the same time point. To analyse differences over time, we performed a mixed-model linear regression of continuous variables and logistic regression for a binary outcome. Mixed effect models were built with random effects for intercept. We considered *P* < 0.05 as significant. Statistical analyses were performed using IBM SPSS Statistics for Windows version 24.0 (IBM Corp, Armonk, NY), and STATA/IC 14.1.

## Results

### Demographic details and baseline data

We included 696 patients at nursing home admission. During the assigned period between January 2012 and January 2015, 189 nursing home patients died, of whom we were able to assess *N* = 134 (70.9%) on the day of death and *N* = 82 (43%) on the day they were perceived as dying (Fig. 1).

Tables 1, 2, 3 and 4 present further details on the participants and the results of the analyses. Eighty-two (61%) participants were women, and the mean age was 85.9 years (SD 6.3) (Table 1). Among the patients included on the day of death, 88 patients (68%) had moderate to severe dementia, 24 (18%) mild dementia, and 18 (14%) no dementia at admission to the nursing home (Table 1).

**Table 1:** Demographic and clinical variables at baseline in the end-of-life care sample (*N* = 134)

	N	Percent or mean	Missing values
Age, mean (SD)	134	85.9 (6.3)	NM
Gender, female (%)	134	82 (61%)	NM
Without dementia (CDR 0, 0.5), %	18	14%	4
Mild dementia (CDR 1), %	24	18%	
Moderate and severe dementia (CDR 2–3), %	88	68%	
RAI-NH, masticatory problems (%)	8	9%	41
RAI-NH, dysphagia (%)	8	9%	41
RAI-NH, oral pain (%)	5	5%	41
MOBID-2 mouth item (0–10), %			5
0–2	116	90%	
3–7	13	10%	
8–10	0	0%	
ESAS appetite (0–10), %			25
0–2	81	74%	
3–7	22	20%	
8–10	6	6%	
No nutrition adaption, %	73	80%	43
Tube feeding, %	0	0%	42
Grinded food, %	6	7%	42
Customised diet, %	9	10%	42

No missing values (NM)

Clinical Dementia Rating (CDR) (low scores indicate low severity of dementia)

Residents Assessment Instrument for Nursing Homes (RAI-NH)

Mobilisation-Observation-Behaviour-Intensity-Dementia-2 Pain Scale (MOBID-2) (scores 0–10, increasing score indicates increasing pain)

Edmonton symptom assessment system (ESAS) (scores 0–10, increasing scores indicate increasing

At admission, the frequencies for pain in the mouth (5%), dysphagia (9%) and masticatory problems (9%) were low as measured with the RAI-NH. Loss of appetite, defined as an ESAS appetite score of 3 or higher, was present in *n* = 28, 26% of the patients, and 20% received nutrition support (Table 1).

### Oral symptoms on the day perceived as dying

Xerostomia (73%), followed by dysphagia (66%) and masticatory problems (57%) were the most frequent oral symptoms on the day the patient was perceived as dying. Two or more oral symptoms were present in 88% of the patients on the day perceived as dying.

### Oral symptoms on the day of death

On the day of death, the most frequent symptoms were xerostomia (66%), dysphagia (59%), masticatory problems (50%) and halitosis (41%). We found that 69% had two or more symptoms, and that 20% had ≥ 6 oral symptoms.

### Mouth care

Oral care was provided to most patients on the day perceived as dying (93%) and on the day of death (89%). However, only 16% received oral care hourly, the same proportion on both the day perceived as dying and on the day of death (Table 2).

**Table 2:** Oral symptoms on the day perceived as dying and on the day of death

Oral symptoms	Day perceived as dying (*N* = 82)	Day of death (*N* = 134)	*P*-value	Missing data day perceived as dying N (%)	Missing data day of death N (%)
Own teeth	47%	52%	0.87	20 (24)	9 (7)
Prosthesis in mouth last 24 h	22%	16%	0.35	20 (24)	19 (14)
Mouth care the last 24 h	93%	89%	0.34	20 (24)	12 (9)
Mouth care frequency, times/day^*^					
once	16%	14%	0.63	23 (28)	29 (22)
3 times	21%	13%			
> 3 times	48%	56%			
Every hour	16%	16%			
Symptoms					
Xerostomia	73%	66%	0.35	18 (22)	12 (9)
Mucus	45%	39%	0.58	19 (23)	12 (9)
Crust	32%	32%	0.64	18 (22)	13 (10)
Halitosis	41%	41%	0.64	18 (22)	15 (11)
Bleedings	9%	9%	0.33	18 (22)	12 (9)
Sore	2%	8%	0.39	18 (22)	12 (9)
Candidiasis	11%	6%	0.44	18 (22)	12 (9)
Other infection	2%	5%	0.44	18 (22)	12 (9)
Pain at rest	9%	6%	0.72	19 (23)	13 (10)
Pain during mouth care	21%	12%	0.20	22 (27)	13 (10)
Masticatory problems	57%	50%	0.44	20 (25)	17 (13)
Dysphagia	66%	59%	0.44	18 (22)	14 (11)
Reduced ability to speak	16%	10%	0.25	18 (22)	14 (11)
Two symptoms or more	88%	69%	< 0.001	26 (32)	23 (17)
Six symptoms or more	16%	20%	< 0.001	26 (32)	23 (17)
Nutrition					
Low fluid intake	84%	78%	0.71	23 (28)	13 (10)
Dehydration	66%	65%	0.26	22 (27)	13 (10)
Low food intake	63%	63%	0.81	22 (27)	13 (10)
Cachexia	48%	41%	0.16	21 (26)	13 (10)

*Proportion of patients who received mouth care

*P*-value from mixed model logistic regression

### Nutrition and impact of care

When investigating the intake of food and drink on the day perceived as dying and on the day of death, we found that most patients drank less than 1 L (84% vs78%), a high proportion had dehydration (66% vs. 65%), while 63% ate less than one meal in the last two days of life and 48% vs. 41% had severe weight loss due to severe illness (cachexia) according to the RAI-NH tool (Table 2).

### Cognitive function

People with dementia at admission experienced more xerostomia on the day of death (73%) than people without dementia at admission (50%), *P* = 0.049. Masticatory problems were also more prevalent on the day of death in people with dementia at admission (56%) compared to people without dementia (32%), *P* = 0.038 (Table 3).

**Table 3:** Oral symptom burden on the day of death broken down by baseline with/without dementia

Oral symptom	Without dementia (*N* = 26)	With dementia (*N* = 107) ^*^	*P*-value
Xerostomia	50%	73%	0.049
Mucus	32%	38%	0.564
Crust	32%	34%	0.868
Halitosis	29%	45%	0.158
Bleedings	0%	10%	0.120
Sore	0%	10%	0.120
Candidiasis	5%	10%	0.421
Other infection	0%	6%	0.236
Pain at rest	9%	8%	0.178
Pain during mouth care	9%	12%	0.677
Masticatory problems	32%	56%	0.038
Dysphagia	45%	63%	0.132
Reduced ability to speak	5%	11%	0.372

*P*-value from Chi-Square

^*^1 missing unable to be diagnosed by the physicians

### Associations between cachexia and oral symptoms

For the logistic regression analysis at baseline, we used the oral symptoms at baseline as independent variables and leaving more than 25% of the food at baseline as a dependent variable. Results show that patients with masticatory problems (OR 4.1 95% CI 1.6–10.4, *P* = 0.003), dysphagia (OR 4.1 95% CI 1.7–10.1, *P* = 0.002) and painful mouth (OR 5.7 95% CI 1.5–21.8, *P* = 0.010) were more likely to experience reduced food intake (leaving more than 25% of their food) at baseline (Table 4).

We built another logistic regression for the day of death using cachexia as a dependent variable and symptoms on day of death as independent variables. The participants identified by the caregivers as having xerostomia (OR 7.4, 95% CI 2.6–21.0, *P* < 0.001), dysphagia (OR 2.3, 95% CI 1.1-5.0, *P* = 0.038) and halitosis (OR 5.2, 95% CI 2.3–11.7, *P* < 0.001) were more likely to have cachexia when they died (Table 4).

**Table 4:** Associations between leaving food at baseline assessment and oral symptoms at baseline, and cachexia on day of death and oral symptoms on day of death

	Baseline	Day of death
	Leaving > 25% of the food*	Cachexia**
Symptoms	OR CI *P*	OR CI *P*
Mastication	4.1 1.6–10.4 0.003	0.6 0.1–2.9 0.558
Dysphagia	4.1 1.7–10.1 0.002	2.3 1.0–5.0 0.038
Mouth pain	5.7 1.5–21.8 0.010	0.2 0.0-1.7 0.130
Xerostomia	N/A	7.4 2.6–21.0 <0.001
Halitosis	N/A	5.2 2.3–11.7 <0.001

Associations are reported using crude Odds Ratio (OR), with a 95% CI.

*Residents’ Assessment Instrument for nursing homes (RAI-NH), leaving 25% of the food (cachexia)

**Residents’ Assessment Instrument for palliative care (RAI-PC)

## Discussion

Our study found that nursing home patients have many oral health problems when they die. People with dementia at admission to a nursing home had more xerostomia and masticatory problems when they died, compared to people without dementia at admission. Cachexia was associated with xerostomia, dysphagia, and halitosis on the day of death. To enhance comfort and oral intake, it is of key importance that the clinician and nursing home staff identify and treat these symptoms during the whole nursing home stay, and increase their attention to detail when the patient is approaching the end of life [30, 31].

Our results on the vast oral symptom burden correspond well with findings from an Italian study which included 75 cancer patients receiving hospice care [32]. The paper described oral health problems and the impact of daily care and found that 75% had xerostomia, 14% pain and 55% of the sample had mucus. Their study further revealed that by introducing a structured assessment, the overall oral symptom severity changed significantly (*P* < 0.0001). We have demonstrated that xerostomia is also the most prominent oral symptom in dying nursing home patients overall. This had previously only been described in patients dying from cancer [33]. Moreover, our finding that 73% had xerostomia on the day perceived as dying and 66% on the day of death, is well in line with findings from a study by Matsuo et al. (2016), who included 46 dying cancer patients and detected that 78% were affected [17]. The finding is not surprising because when death is imminent, the dying patient shifts from nose breathing to mouth breathing, which leads to oral dryness. Reduced salivary gland function, and the underlying diseases are both factors that increase oral symptoms such as xerostomia and infections, including candida albicans.

The thorough description of signs and symptoms exhibited by patients in this study might be useful for helping other caregivers to detect the total symptom burden and identify dying in persons with dementia. A comprehensive description of symptoms on the day perceived as dying and the day of death is vital in order to provide the right treatment. To keep the mucosa and tongue clean and moist, the use of humidifiers and lubricants is recommended. The cleaning frequency, structured assessment, planned treatment and evidence-based care pathways are therefore key elements. In conscious patients still able to swallow, sips of water, regular mouthwash without alcohol, or chewing gum (not for persons with dementia) and sweets with xylitol have been recommended [34, 35]. Considering the severe oral symptom burden in nursing home patients with dementia, the study by Kvalheim et al. (2019) is especially interesting. They investigated the effect of glycerol 17%, Aequasyal, and Salient on 30 patients recruited from two palliative care units in Norway, and the results demonstrate that glycerol had the best effect on relief from xerostomia directly after application, but had no effect after 2 h. Aequasyal and Salient had a longer effect, but they were not the preferred choice for the patients because of the disagreeable taste of Aequasyal and the unpleasant, sticky consistency of Salient. These findings revealed a necessity for mouth care and lubricant application with a minimum frequency of once every 2–3 h [36].

Our study found that most patients received oral care during their last 24 h, and 3 out of 4 had oral care more than 3 times per day or hourly. Ezenwa et al. (2016) state that caregivers recognize their responsibility regarding patients’ oral hygiene, but do not assess oral symptoms frequently in dying patients [37]. A study from Taiwan found that nurses and nursing assistants lack knowledge in palliative oral care for advanced dementia. However, the knowledge was higher among educated and experienced staff [38]. Continuous education that incorporates oral care competence is therefore very important.

There is currently no validated assessment tool to identify the broad range of oral symptoms in dying patients with dementia. We believe that it is well justified to develop the standard of oral care for dying nursing home patients, with clear protocols for assessment, cross-professional treatment, reassessment after interventions, and documentation [5, 39]. Interestingly, it was possible for the nursing home staff in our study to identify the various symptoms and hence vital for staff to do so. The Oral Health Assessment Tool (OHAT) for Dental Screening is available in Norwegian and a very good tool for the general nursing home population [10]. However, according to our result it is lacking information on symptoms relevant for the dying old such as ability to speak, other symptoms need to be very severe to be assessed as relevant and placed under another heading, such as for halitosis. The large variation of symptoms included in the various assessment tools are also recently addressed by a scoping review finding a variation from 2 until 20 different symptoms [18].

Decayed teeth in people with dementia can be caused by polypharmacy, especially with the use of anticholinergic drugs such as antidepressants, medication related to Parkinson’s disease, and pain medication causing xerostomia [40]. There is a direct link between xerostomia and dehydration, malnutrition and infections in the oral cavity. When death is imminent, distressing symptoms are often managed by drugs with anticholinergic side effects, such as morphine for pain relief, benzodiazepines to treat anxiety, or diuretics in case of dyspnea [13, 16].

In the last days and hours of life, comfort and reduced physical and mental distress are the focus of care, and important factors to ensure dignity [41]. Expression of own needs and wishes to caregivers and the important last communication with relatives requires a clean, fresh and moist mucosa and oral cavity with functioning dentures [42]. Dysphagia and xerostomia will restrict the patients’ ability for oral intake and communication. Proper mouth care is described as an important part of palliative care in general. However, this has been better investigated in dying cancer patients compared to people with dementia. In fact, according to Kvalheim et al. (2015), only 44% of nursing home staff recognized that oral health problems are of importance in dying patients. The study further concluded that patients are exposed to a great number of oral procedures without established efficacy [14].

### Limitations to our study

We have not been able to provide information on the oral status of the included sample of participants in our study except for the percentage with their own teeth, hence we lack vital information on dental status. Situations such as broken teeth or inflamed gum and other events might introduce symptoms such as pain. Our analysis found consistency in oral symptoms between the day perceived as dying and the day of death. However, a low sample size might have introduced a type I error where meaningful effects might not be detected due to low power. The sample size varies between the assessment point of the day perceived as dying and the day of death. We therefore used a mixed model analysis to address individualized changes over time and be resilient to sample size variation. Our study lacks information on the specific oral cleaning procedures used, and this limits our ability to comment on the total treatment plan provided and its efficacy. The *P*-value for the difference in xerostomia in people with or without dementia at baseline was *P* = 0.049, so the null hypothesis was almost rejected. In a larger sample, the evidence might be stronger. These questions need to be addressed in future studies. Another limitation is the possible reporting bias that can be introduced when interviewing caregivers who are directly involved in day-to-day care of the patients. However, the data obtained does not indicate this.

A further limitation is the use of an unvalidated tool for oral assessment of people with dementia and unconscious, dying, nursing home patients. However, currently no comprehensive tool has been developed to identify oral symptoms in dying nursing home patients with dementia. Future tools need a higher degree of accuracy when symptoms are assessed to provide higher validity than was possible with the tools used in our study, thus leading to some inconclusiveness in the statistical tests.

Pain assessment encompasses multiple methodologies, with self-report measures, notably the unidimensional Numeric Rating Scale (NRS), being predominant. Despite the widespread acknowledgment of self-reports as the gold standard in pain assessment, their applicability is limited when an individual is unable to communicate pain effectively. Consequently, behavior-based pain assessment tools have been developed as alternatives. Reliance solely on any singular tool for evaluating pain and treatment efficacy is insufficient; incorporating clinical judgment and striving for self-report, where possible, remains essential.

In conclusion, the magnitude and severity of identified oral symptoms highlights the substantial need for systematic assessment and oral care, especially in people with dementia, during the entire nursing home stay and particularly at the end of life. Palliative oral care needs accurate measurements that are valid and reliable, and being such a complex task, cross-professional competence is required. Our findings underline the need for evidence-based oral palliative assessment and care, valid guidelines, and improved training for nursing home staff. To undertake this is it important to include oral health care professionals in the team and as important professionals in the nursing homes.

## Data Availability

Datasheets and data files contain information that makes them ineligible for publication. However, they may be retrieved from the corresponding author in collaboration with the primary investigator, Sverre Bergh, on request.
